# Phenotypic Variability of a Pathogenic *PKP2* Mutation in an Italian Family Affected by Arrhythmogenic Cardiomyopathy and Juvenile Sudden Death: Considerations From Molecular Autopsy to Sport Restriction

**DOI:** 10.3389/fcvm.2021.635141

**Published:** 2021-05-20

**Authors:** Maria Pia Leone, Pietro Palumbo, Johan Saenen, Sandra Mastroianno, Stefano Castellana, Cesare Amico, Tommaso Mazza, Domenico Rosario Potenza, Antonio Petracca, Marco Castori, Massimo Carella, Giuseppe Di Stolfo

**Affiliations:** ^1^Division of Medical Genetics, Fondazione IRCCS Casa Sollievo della Sofferenza, San Giovanni Rotondo, Italy; ^2^Department of Cardiology, University Hospital Antwerp, University Antwerp, Antwerp, Belgium; ^3^Cardiovascular Department, Fondazione IRCCS Casa Sollievo della Sofferenza, San Giovanni Rotondo, Italy; ^4^Bioinformatic Unit, Fondazione IRCCS Casa Sollievo della Sofferenza, San Giovanni Rotondo – Istituto Mendel, Rome, Italy

**Keywords:** novel PKP2 mutation, arrhythmogenic cardiomyopathy, juvenile sudden death, molecular autopsy, sport restriction

## Abstract

**Background:** Arrhythmogenic cardiomyopathy (ACM) is a genetic disorder with an estimated prevalence between 1:2,000 and 1:5,000 and is characterized by the fibrofatty replacement of cardiomyocytes that predisposes to malignant arrhythmias, heart failure, and sudden cardiac death. The diagnosis is based on the 2010 Task Force Criteria including family history, electrocardiographic traits and arrhythmogenic pattern, specific gene mutations, and structural and/or histological abnormalities. Most ACMs display an autosomal dominant mode of inheritance often with incomplete penetrance and variable expressivity. Genetic screening of patients with ACM identifies pathogenic or likely pathogenic variants, prevalently in genes encoding the cardiac desmosome (*PKP2, DSP, DSC2, DSG2*, and *JUP*) or less frequently in non-desmosomal genes (*CTNNA3, PLN, TMEM43, RYR2, SCN5A, CDH2*, and *DES*).

**Methods:** In the present study, we performed molecular autopsy in a boy who died suddenly during physical exertion. In addition to post-mortem examination, a DNA sample was analyzed with next-generation sequencing (NGS).

**Results:** The genetic analysis revealed the presence of pathogenic heterozygous c.314del (p.Pro105Leufs^*^7) frameshift variant in the PKP2 gene. Cascade screening of family members allowed us to identify 12 mutation carriers and to intervene on subjects at risk, many of whom were athletes.

**Conclusions:** Molecular autopsy can establish cardiogenetic diagnosis and allow appropriate preventative measures in high-risk relatives.

## Introduction

Arrhythmogenic cardiomyopathy (ACM) is a genetic disorder with an estimated prevalence between 1:2,000 and 1:5,000 and is characterized by the fibrofatty replacement of cardiomyocytes (1). The progressive fibrotic replacement causes electrical instability with an increased risk of syncope and sudden cardiac death (SCD), or alteration of cardiac function with right- or bi-ventricular heart failure resembling dilated cardiomyopathy (2). The diagnosis is challenging and based on the conjugate of 2010 Task Force criteria including family history, peculiar electrocardiographic, arrhythmic, and structural and/or histological abnormalities (3). Inheritance of ACM is classically considered autosomal dominant; however, incomplete penetrance and highly variable and age-dependent disease expression are observed (4). Rarely, autosomal recessive inheritance is found in ACM both with or without cutaneous involvement.

Genetic screening of patients with ACM identifies pathogenic or likely pathogenic variants, predominantly in genes encoding the cardiac desmosome (*PKP2, DSP, DSC2, DSG2*, and *JUP*) or less frequently in non-desmosomal genes (*CTNNA3, PLN, TMEM43, RYR2, SCN5A, CDH2*, and *DES*) (5).

The *PKP2* gene encodes a member of the Armadillo (ARM) repeat proteins. Plakophilin proteins contain numerous ARM repeats, localize to cell desmosomes and nuclei, and participate in linking cadherins to intermediate filaments in the cytoskeleton (6). Plakophilin-2 is important for the assembly of junctional proteins and represents an essential morphogenic factor and architectural component of the heart (7).

Mutations in the *PKP2* gene, whose gene–phenotype relationships are available in Online Mendelian Inheritance in Man as OMIM^*^602861, encoding the main cardiac plakophilin, at locus 12p11, represent 10–45% of the genotyped ACM patients (1).

In the present study, we performed molecular autopsy in a boy who died suddenly during physical exertion. In addition to *post-mortem* examination, a DNA sample was analyzed by NGS. The genetic analysis revealed a pathogenic heterozygous frameshift variant in the *PKP2* gene (c.314del;p.Pro105Leufs^*^7). Cascade screening of 19 family members allowed us to identify 12 mutation carriers and to intervene on subjects at risk, many of whom were athletes.

## Case Report

**Figure 1:**
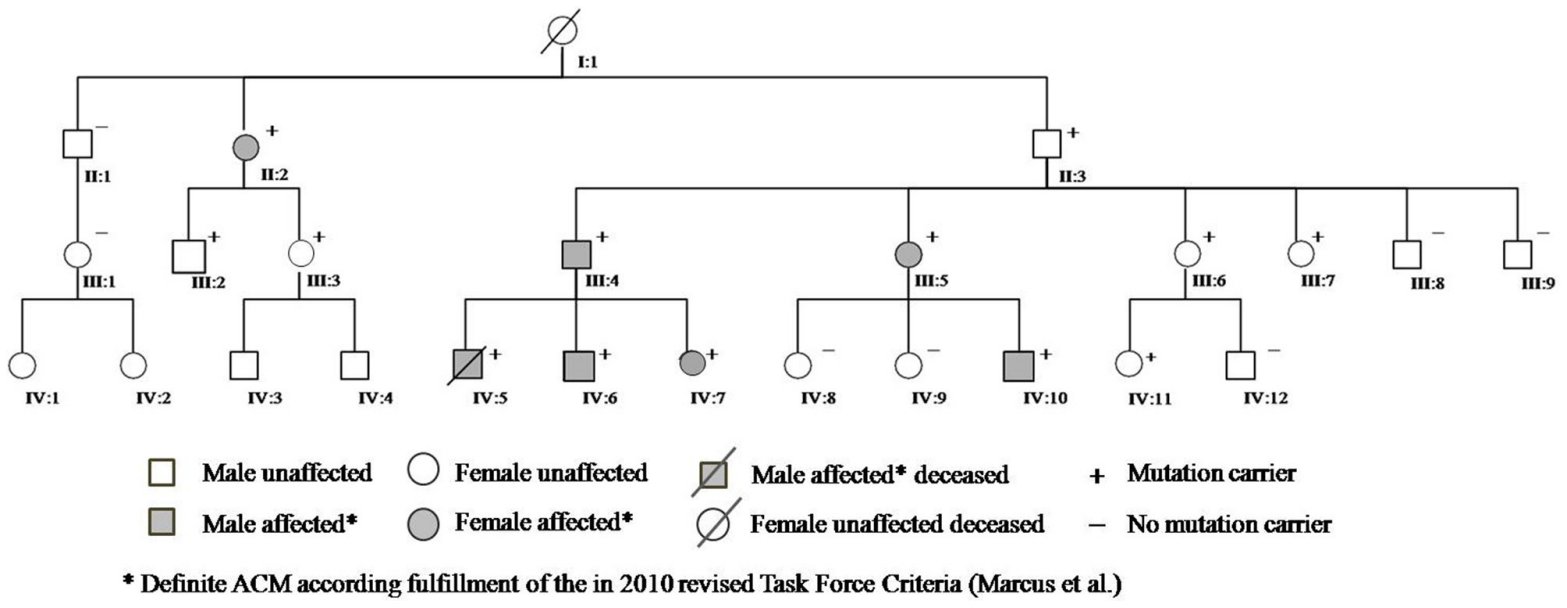
Family pedigree.

A 13-year-old boy (Figure 1, IV:5) without prior medical history died suddenly during an hour of physical education following unsuccessful cardiopulmonary resuscitation, despite evidence of ventricular fibrillation treated by external defibrillation. Autopsy was requested and initial pathology report concluded for dilated cardiomyopathy with intramural fibrosis.

The parents, fearing for their other two children, underwent cardiologic evaluation at our institution. They reported that their son had complained of palpitations in the month before his death; after adequate information and psychological support for the bereavement process, they consented to genetic testing and molecular autopsy on their son's biological material. NGS analysis revealed a novel frameshift variant in the *PKP2* gene (c.314del;p.Pro105Leufs^*^7). The *PKP2* gene encodes plakophilin, which has been implicated in literature with arrhythmogenic cardiomyopathy (ACM). Therefore, we asked the family for permission to review the autoptic examination in light of the molecular data, a more thorough second analysis was consistent with ACM with biventricular involvement and fibrofatty replacement in both ventricles, supporting the diagnosis suggested by the genetic analysis. At this stage, adhering to the ACMG standards and guidelines and in the absence of segregation and functional data, this *PKP2* variant was classified a class 4 “likely pathogenic” variant warranting further co-segregation analysis and predictive testing in the first-degree relatives (FDRs) (8).

**Figure 2:**
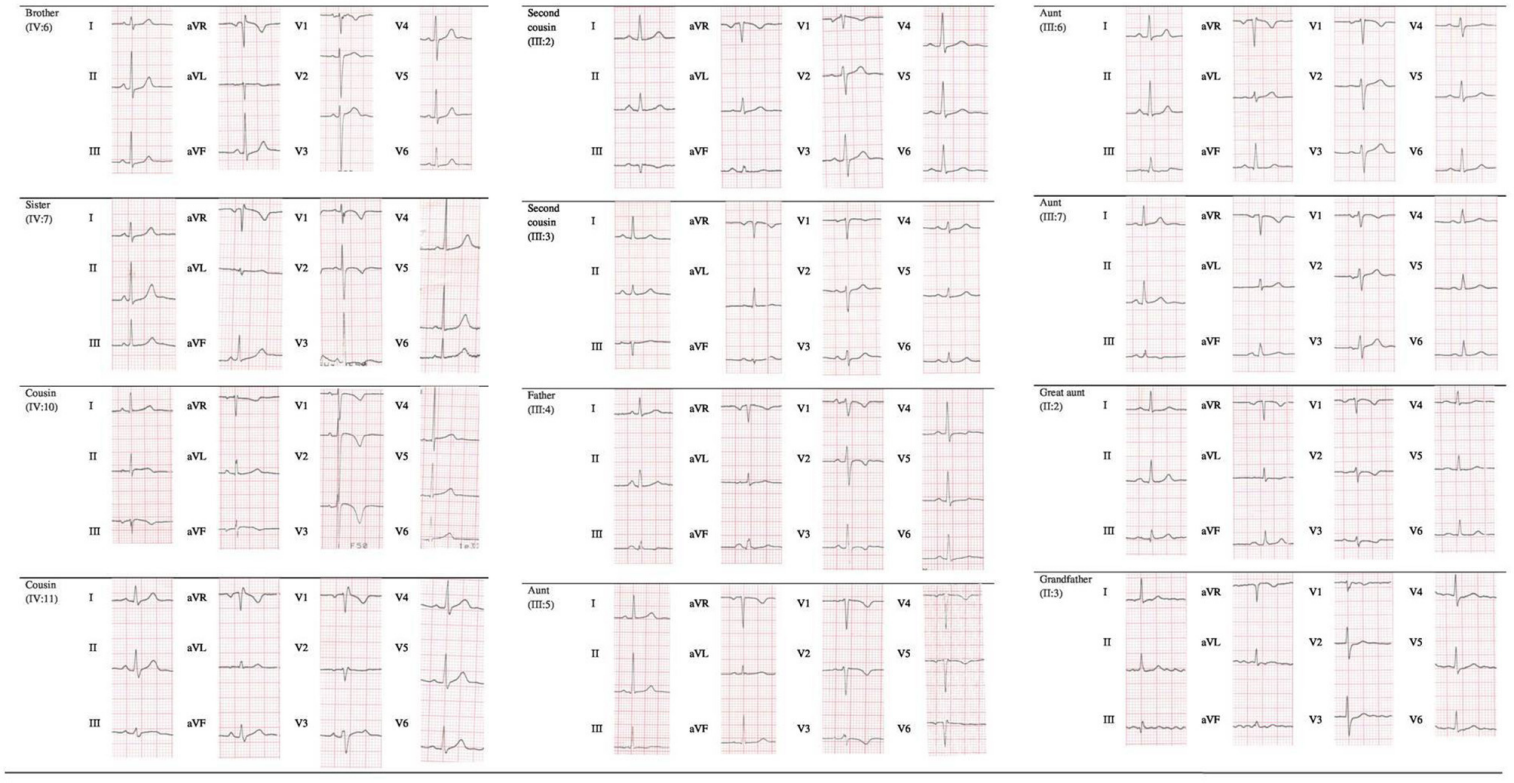
Electrocardiogram (ECG) finding in gene carriers.

Cascade screening of family members was subsequently initiated to evaluate possible carriers of the same mutation and identify other patients at risk with asymptomatic or unrecognized cardiomyopathy (Table 1). The first to be studied was his 42-year-old father (III:4) who reported palpitations. ECG evaluation revealed T wave inversion in V1–V3, minimal prolonged terminal activation duration (TAD) in V1, fragmented QRS in inferior leads, low QRS-voltage (Figure 2) and Holter recording showed frequent ventricular premature complexes (2388 PVCs/24 h) with two different morphologies (right bundle branch block and left bundle branch block pattern). Echocardiography showed enlargement of the right ventricular outflow tract (RVOT) (PLAX 41 mm and PSAX 36 mm, respectively). Cardiac magnetic resonance imaging highlighted the dilated right ventricle with contractile dysfunction (RVEF 35%, RVEDVi 105 mL/m^2^) and hypokinesia of the mid to apical free wall segments, fatty infiltration within the free wall of the right ventricle, and minimal adipose substitution in the epicardium of the apical left ventricle. A coronary angiography ruled out critical coronary stenosis. According to the 2010 Task Force Criteria, “definite” ACM was diagnosed in this patient for the presence of inverted T waves, prolonged TAD, and sudden death of 13-year-old son, and therapy with beta-blocker and ACE inhibitors was initiated. ACM mortality risk was estimated as 32.7% at 5 years, and an ICD was implanted for primary prevention (9–11).

**Table 1:** Clinic and genetic features of family members.

Family member^*^	**Sex**	**Age at first evaluation ys**	**PKP2 mutation carrier**	**Physical activity**	**Symptoms**	**ECG**	**HR bpm**	**QRS msec**	**Echocardiogramm**	**Teadmill test**	**24 h Holter monitoring**	**cMR**	**Diagnosis of ACM according to 2010 TFC**	**Management**
Third cousin (IV:1)	F	16	n.p.	competitive volleyball									-	
Third cousin (IV:2)	F	13	n.p.	competitive volleyball									-	
Third cousin (IV:3)	M	15	n.p.	competitive volleyball									-	
Third cousin (IV:4)	M	15	n.p.	competitive volleyball									-	
Proband (IV:5)	M	_	yes	amateur football	SD at 13 ys (histology specimen: fibrofatty replacement)	_	_	_	_	_	_	_	Proband	_
Brother (IV:6)	M	11	yes	amateur football	no	epsilon wave in V1	68	82	small ventricular trabeculations	_	93 PACs (82 couplets and 72 short runs); 1 PVC	_	Definite ACM−2 Major TFC: Epsilon wave, confirmed histopathology in brother (IV:5)	close follow-upphysical activity forbidden
Sister(IV:7)	F	8	yes	no	palpitations	TWI V1,V2,V3	68	74	small ventricular trabeculations	_	386 PACs (222 couplets and short runs of max 12 beats); 163 PVCs (1 triplet with LBBB morphology with horizontal axis)	_	Definite ACM−2 Major TFC: T- wave inversion in V1-3, confirmed histopathology in brother (IV:5)	close follow-upphysical activity forbiddenpropanolol
Cousin (IV:8)	F	21	no	no	no	normal	87	74	Normal	_	_	_	Absent	_
Cousin (IV:9)	F	20	no	no	no	normal	66	82	Normal	_	_	_	Absent	_
Cousin (IV:10)	M	11	yes	amateur football	no	fragmented QRS in D2 and aVF;ST elevation in D2 and aVF; TWI V1,V2,V3	47	90	PLAX RVOT: 36.5 mm (PLAX/BSA: 24 mm/m^2^); PSAX RVOT: 35 mm (PSAX/BSA: 23mm/m^2^); e'14 cm/sec; TAPSE 24.7 mm	6 PVCs during stress with LBBB morphology	0 PAC; 50 PVCs with LBBB morphology	subepicardial fibrosis in inferior and later LV wall	Definite ACM−2 Major TFC: T- wave inversion in V1-3, pathogenic genotype	close follow-upphysical activity forbidden
Cousin (IV:11)	F	17	yes	amateur volleyball	panic attacks	TAD 60 ms	78	98	TAPSE 25 mm; e' 20 cm/sec; FAC 50%	0 PAC;0 PVC	0 PAC; 1 PVC	_	Borderline ACM−1 Major and 1 Minor TFC: pathogenic genotype, prolonged TAD	close follow-upphysical activity forbidden
Cousin (IV:12)	M	13	no	amateur football	no	incomplete RBBB	90	98	apical RV hypokinesia; PLAX RVOT: 31.5 mm (PLAX/BSA: 16 mm/m^2^); PSAX RVOT: 37.4 mm; TAPSE 24 mm	_	_	normal	Absent	_
Second cousin(III:1)	F	48	no	amateur volleyball	no	incomplete LBBB	63	70	TAPSE 29 mm, e' 13 cm/sec	n.p.	n.p.	_	Absent	
Second cousin(III:2)	M	49	yes	several amateur sports	no	incomplete RBBB;	62	88	regional RV hypokinesia; PSAX RVOT 37 mm	n.p.	n.p.		Possible ACM−1 Major TFC: pathogenic genotype. RVOT enlargement and no akinesia, no dyskineasia	close follow-up
Second cousin(III:3)	F	49	yes	several amateur sports	no	low QRS-voltage	75	74	apical RV hypokinesia; PSAX RVOT: 38 mm	n.p.	n.p.	_	Possible ACM−1 Major TFC: pathogenic genotype. Echo shows only RVOT enlargement and no akinesia or dyskineasia	close follow-up
Father(III:4)	M	42	yes	no	no	fragmented QRS in D2 and aVF;TAD 60 msec; QRS complex > 110 msec in V1;TWI V1-V4;low QRS-voltage	59	88	PLAX RVOT: 41 mm; PSAX RVOT: 36 mm	n.p.	40 PACs; 10 PVCs with RBBB and LBBB morphologies	dilated RV with contractile dysfunction; middle and apical free wall RV hypokinesia;adipose substitution in apical LV;fatty infiltration in free wall RV.	Definite ACM−2 Major and 1 Minor TFC: pathogenic genotype, confirmed histopathology in son (IV:5), T wave inversion V1-3, TAD 60ms (only 2 Major TFC attributed, since genotype and FDR histopathology are from the same criterion group)	close follow-upbisoprolol;ramipril;ICD implantation
Aunt(III:5)	F	41	yes	no	palpitations	TWI V1-V5	75	94	apical RV hypokinesia; PLAX RVOT: 33 mm; PSAX RVOT: 36 mm	n.p.	0 PAC; 0 PVC	dyskinesia of diaframmatic wall RV.	Definite ACM−3 Major TFC: pathogenic genotype, T wave inversion V1-5, Dyskinesia with PLAX 33 mm	close follow-upnebivolol
Aunt(III:6)	F	39	yes	no	palpitations		73	86	free wall RV hypokinesia; PLAX RVOT: 33 mm	n.p.	11 PAC; 3 PVC	_	Possible ACM−1 Major TFC: pathogenic genotype, PLAX 33 mm	close follow-up
Aunt(III:7)	F	34	yes	no	palpitations	fragmented QRS in D3 and aVF	97	76	Normal	0 PAC;0 PVC	n.p.	normal	Possible ACM−1 Major TFC: pathogenic genotype	close follow-upbisoprolol
Uncle(III:8)	M	37	no	several amateur sports	no	normal	65	90	TAPSE 32 mm	_	_	_	Absent	
Uncle(III:9)	M	34	no	amateur multisport	no	incomplete RBBB	72	104	TAPSE 31 mm	_	_	_	Absent	
Great uncle (II:1)	M	78	no	no	no	fragmented QRS in D2, D3 and aVF;low QRS-voltage	68	82	TAPSE 25 mm; e' 16 cm/sec	_	_	_	Absent	
Great aunt (II:2)	F	71	yes	no	angina	TWI V1-V3;fragmented QRS in V3;low QRS-voltage	73	64	free wall RV hypokinesia; PLAX RVOT: 33 mm; PSAX RVOT: 36 mm	_	0 PAC; 0 PVC	_	Definite ACM−2 Major TFC: T wave inversion V1-3, pathogenic genotype	close follow-upcarvedilol increase
Grandfather (II:3)	M	70	yes	no	no	persistent AF; fragmented QRS in aVF and V1; TAD in V1;low QRS-voltage	96	80	LA enlargement; TAPSE 25 mm	_	_	_	Possible ACM−1 Major TFC: pathogenic genotype	close follow-up
Great grandmother (I:1)	F	_	n.p. *However, obligate carrier*	no	SD at 62 ys (two cousins died suddenly at 16 and 18 years)								Possible ACM−1 Major TFC: Obligate carrier pathogenic genotype	

*AF, Atrial fibrillation; ARVC, arrhythmogenic right ventricular dysplasia; AVB, atrioventricular block; cMR, cardiac magnetic resonance imaging; F, female; HR, heart rate; LA, left atrium; LBBB, left bundle branch block; LV, left ventricle; M, male; n.p., not performed; PAC, premature atrial contraction; PVC, premature ventricular contraction; RBBB, right bundle branch block; SD, sudden death; TAD, terminal activation duration; TFC, Task Force Criteria; TWI, T wave inversion*.

* *Roman and Arabic numerals correspond to generation and pedigree number shown in Figure 1*.

Genetic evaluation revealed the presence of the same PKP2 variant that was deemed responsible for the sudden cardiac death of his son. This evidence of co-segregation of the genotype with the ACM phenotype allows re-classification of the PKP2 variant to a class 5 pathogenic mutation and further strengthening the need for predictive testing in other relatives of this family (8).

Cascade clinical and genetic screening was initiated with 19 relatives contacted in total; 12 additional subjects were found to be genotype positive and six of them diagnosed with two or more major TFC criteria; hence, definite ACM; five of mutation-positive individuals used to play high-intensity sports. Moreover, his paternal great-grandmother (I:1), who had died suddenly several years ago without apparent cause at the age of 62, and her two cousins known with sudden death at 16 and 18 years of age, involved in high-level sport activity (volleyball), were all likely affected.

Apart from genetic screening, the most prominent clinical traits found in the *PKP2* mutation carriers were the ECG abnormalities, characterized by T wave inversion beyond V2 in the absence of intraventricular conduction disturbances, QRS fragmentation, epsilon wave, and TAD equal or longer than 55 ms, as shown in Table 1 and Figure 2. Echocardiographic abnormalities were less prevalent.

Depending on the individual findings, preventative measures were taken including medical therapy with beta blocker and lifestyle modification. According to current consensus, we forbade competitive sport in genotype-positive individuals and phenotypically affected relatives. However, to preserve psychological health and reduce the burden of intrusive lifestyle alterations, cardiogenetic counseling was done in conjunction with our psychologist team. In children, a gradual retreat from sport participation was imposed, and parents were advised to replace it with artistic activities, like theater and music performance.

### Library Preparation and Next-Generation Sequencing

A Cardiac Disease Sequencing Panel involving 76 genes (see Table 2 in the Supplements), including genes related to hypertrophic cardiomyopathy, dilated cardiomyopathy, and arrhythmogenic cardiomyopathy, was designed according to data obtained from scientific literature. Probes were designed using Agilent SureDesign Custom design tool (https://earray.chem.agilent.com/suredesign/:) the regions of interest (ROI) for this panel included all exons plus 25-bp flanking intron regions. The total amplicon number was 19.648, and the target size was 366.196 kb with a theoretical coverage of 99.32% for our targeted regions.

**Table 2:** List of analyzed genes.

ABCC9	JUP	PRKAG2
ACTC1	KCNE3	PTPN11
ACTN2	KCNH2	RAF1
ANKRD1	KCNJ2	RBM20
BAG3	KCNQ1	RYR2
CACNA1C	LAMA4	SCN10A
CACNB2	LAMP2	SCN1B
CASQ2	LDB3	SCN3B
CAV3	LMNA	SCN4B
CHRM2	MURC	SCN5A
CRYAB	MYBPC3	SGCD
CSRP3	MYH6	SNTA1
DES	MYH7	TAZ
DOLK	MYL2	TCAP
DSC2	MYL3	TGFB3
DSG2	MYLK2	TMEM43
DSP	MYOM1	TNNC1
DTNA	MYOZ2	TNNI3
EMD	MYPN	TNNT2
FHL2	NEBL	TPM1
GATAD1	NEXN	TRDN
GLA	PDLIM3	TTN
GPD1L	PKP2	TTR
ILK	PLN	VCL
JPH2	PRDM16	

The DNA of the patient was isolated from biological material. Peripheral blood samples were taken from his parents, and genomic DNA was isolated by using Bio Robot EZ1 (Quiagen, Solna, Sweden). A library of all coding regions of the 76 genes was obtained using the Haloplex target enrichment kit according to the manufacturer's instructions (Agilent Technologies, Santa Clara, CA, USA). At last, the enriched DNA fragments were sequenced on an Illumina MiSeq platform (Illumina, San Diego, CA, USA) using a MiSeq Reagent kit V3 300 cycles flow cell (Illumina, San Diego, CA, USA).

### Bioinformatics Analyses

The produced raw paired-end reads underwent quality checking by using the FastQC tool (12) and then aligned to the hg19 reference genome sequence by means of Bowtie (13). Depth of coverage statistics for the target regions were calculated by TEQC ver. 3.47 (14).

Variants were called by means of the HaplotypeCaller tool of GATK ver. 3.8 (15), while functional annotation was carried out by ANNOVAR tool, using the NCBI RefSeq gene and transcript annotation system (updated to January 2018) (16). Variants were checked for their presence in public databases, such as dbSNP ver. 150 (17), ExAC ver. 0.3 (18), and Exome Variant Server (http://evs.gs.washington.edu/EVS, accessed at December 2017), HRC (19) Kaviar (20) and ClinVar (21). Predictions of functional consequences for missense variants were further collected by querying the dbNSFP ver. 3.5 resource and retrieving pre-computed pathogenicity predictions and evolutionary conservation measures (22).

### Sanger Sequencing

The presence of the detected variant was confirmed by gold standard Sanger sequencing. A PCR was carried out to amplify the exon 2 of the *PKP2* gene (NM_001005242) including the variant site, and the PCR product was purified by ExoSAP-IT PCR Product Cleanup Reagent (Thermo Fisher Scientific, Waltham, MA, USA) first, then sequenced on an ABI Prism 3100 Genetic Analyzer (Thermo Fisher Scientific Waltham, Massachusetts, USA) using BigDye Terminator v1.1 sequencing kit (Applied Biosystems, Foster City, CA, USA).

## Results

NGS analysis identified a heterozygous frameshift variant in the *PKP2* gene (NM_001005242:exon2:c.314del;p.Pro105Leufs^*^7) in the patient (IV:5) and his father (III:4), detected with a depth of coverage of 127X.

The variant, reported as pathogenic in ClinVar (rs794729121), causes a shift in reading frame starting at codon proline 105 changing it to leucine, and creating a premature stop codon at position seven of the new reading frame, denoted p.Pro105Leufs^*^7.

The presence of the detected variant was assessed in the patients and in their parents by Sanger sequencing. Furthermore, after the genetic result of the patient (IV:5) and the father (III:4), we performed segregation analysis on other available relatives (II:1; II:2; II:3; III:1; III:2; III:3; III:5; III:6; III:7; III:8; III:9; IV:6; IV:7; IV:8; IV:9; IV:10; IV:11; IV:12). The sequencing analysis revealed further 11 carriers of the reported variant, nine (II:2; III:2; III:3; III:5; III:6; IV:6; IV:7; IV:10; IV:11) with electrocardiographic and/or echocardiographic signs of ACM, and two (II:3; III:7) without detectable ACM traits (Table 1 and Figure 2) conferring a clinical penetrance of 84% (11 with ACM expression out of 13 carriers) with variable expressivity. The estimated 5-year risk for lethal events among the mutation carriers ranges up to 32.7% (as calculated for III:4, according to ARVC calculator) justifying a holistic preventative approach adapted to the individual risk profile of each relative.

A single molecular autopsy in one proband resulted in the tailored improvement of the clinical management in 12 relatives ranging from close cardiac follow-up, lifestyle modification and medication, while immediate ICD implantation was warranted in one high-risk individual (III:4) to prevent a detrimental outcome.

## Discussion

Here we report a 13-year old boy who died suddenly during training despite the readily performed resuscitation maneuvers. NGS sequencing analysis helped physicians to establish the *post-mortem* diagnosis of arrhythmogenic cardiomyopathy by the finding of a pathogenic mutation in the *PKP2* gene. Clinical diagnosis of ACM may be challenging, and therefore, predictive genetic testing may offer valid means to help and identify subjects at risk, before the presence of phenotypical traits, before the onset of early warning symptoms, and ultimately prior to malignant arrhythmic events (23–25). In this case, the discovery of a putative disease-causing variant in the *PKP2* gene raised doubts about the initial cause of death reported in the first autopsy and helped us convince the family to consent to reassessment of the autopsy findings. Today, thanks to contemporary advances in DNA sequencing technologies, *post-mortem* genetic analysis can play an important role in the correct diagnosis of hereditary cardiac disease allowing critical review and interpretation of autopsy findings.

To date, the ClinVar database (26) provides a total of 1,005 annotated PKP2 variants (last accessed November 2020); roughly 65% are linked to cardiac conditions, the remaining 35% has been submitted without an associated clinical diagnosis (27). The variant reported here is a frameshift variant (NM_001005242:exon2:c.314del:p.Pro105Leufs^*^7) expected to result in either a truncated protein product or haplo-insufficiency through nonsense-mediated mRNA decay. Frameshift variants in *PKP2* are strongly associated with ACM, and over the years, other frameshift mutations in the PKP2 gene have been reported in literature (28).

Lack of plakophilin-2 or incorporation of mutant plakophilin-2 in the cardiac desmosomes impairs cell–cell contacts and, as a consequence, disrupts adjacent cardiomyocytes, particularly in response to mechanical stress or stretch, providing a potential explanation for the high prevalence of the disorder in athletes, the frequent occurrence of ventricular tachyarrhythmias and sudden death during exercise, and the predominant affection of the right ventricle (29). In this family, the proband suffered SCD during physical education at school, and two more distant cousins died during a game of volleyball during adolescence underlining the importance of lifestyle modification as first-line preventative measure.

Beyond the NGS approach, segregation analysis represents a powerful methodology to further assess the putative correlation between genotype and phenotype, mainly for variants of uncertain significance (VUS), and to further strengthen the disease-causing probability of likely pathogenic variants. In this family, genetic screening detected the mutation in another 11 subjects following an autosomal dominant inheritance pattern, as reported in literature for other mutations in *PKP2* (4).

This concept highlights the importance of performing family screening, beyond the index case.

Here, we were able to establish a genotype–phenotype correlation in 12 additional informative individuals, which is a very strong evidence that this *PKP2* variant is the predominant cause for the ACM phenotype displayed in this family.

Furthermore, cascade screening allows the identification of other ACM patients at risk and enables individualized preventative measures. Clinical examination showed heterogeneous phenotypic expression, mainly represented by typical electrocardiographic alterations.

Although echocardiographic findings and cardiac MRI examination were not diagnostic in most cases, ECG abnormalities should raise suspicion for the presence of arrhythmogenic cardiomyopathy, in particular, in the precordial leads, characterized by T wave inversion beyond V2, epsilon wave, prolonged TAD (≥55 ms), and QRS fragmentation. In our patients, prevalence of akinesia/dyskinesia was rare; it should be hypothesized that early carrier identification is allowed in the initial ACM stage, without overt phenotype, permitting a better primary care prevention.

Based on the individual clinical characteristics, close follow-up, tailored lifestyle modifications, and medical treatments were advocated. Our young proband carried out physical activity regularly, playing soccer three times a week, a condition that may promote disease expression and progression. In contrast, none of the affected relatives reported being aware of a pre-existing cardiac condition that can progress and exacerbate lethal arrhythmias due to strenuous exercise as many of them were participating regularly in sport activities without any screening. This finding underlines the importance of pre-participation evaluation by electrocardiographic screening integrated with clinical and familiar history for sport-related sudden death prevention (30).

Moreover, according to recent recommendations for participation in competitive and leisure time sports in athletes with cardiomyopathies, athletes who have unequivocal or probable diagnosis of ACM or are genetic carriers of pathogenic desmosomal mutations (even in the absence of phenotypic expression of the disease), should not participate in competitive sports (Class IIa, Level C); these athletes should be advised to limit their exercise programs to leisure-time activities and remain under close clinical surveillance (31). In this perspective, as several family members performed both amateur and competitive sport, in many of them, the diagnosis of ACM represented a reason to suspend these activities.

The psychological reaction to cardiomyopathy diagnosis, in particular, during childhood or adolescence, could have important implications when advocating lifestyle modifications according to the disease state and when confronted with the latent risk to develop disease progression and sudden cardiac death. It is important to identify and address potential psychological suffering early and to incorporate psychological support into the cardiogenetics care team. Patients may experience feelings of anxiety and depression following the diagnosis of an inherited cardiomyopathy that is deemed responsible for the loss of a close relative. The initial proposal for treatment and lifestyle modification can be negotiated and adapted based on the disease stage and patient belief and level of acceptance, allowing intense cooperation between the patient, family, and the healthcare professionals (32).

Our study shows that molecular autopsy is an essential tool in autopsy-negative cases to help establish the cause of death and enable familial screening for other individuals at risk. Such analysis requires highly specialized techniques and trained specialists including geneticists, cardiologists, counselors and psychologists, and should therefore be performed in multi-disciplinary-dedicated cardiogenetics clinics that are experienced in managing hereditary cardiac diseases and cases of sudden death in the young (33).

Healthcare policy makers and cardiovascular societies should support the notion of specialized cardiogenetic hubs that operate in a wider network and be accessed more easily by peripheral centers to permit the extensive cardiogenetic evaluation of these particular SCD cases that may have far-reaching implications for often unknowing relatives and for better understanding of inherited cardiac disorders (34).

## Conclusion

We aim to highlight the importance of molecular autopsy in unsolved sudden cardiac death cases as it may help establish the correct diagnosis, allow the identification of other relatives at risk, and start preventative measures where appropriate. In this case, a single molecular autopsy revealed a highly penetrant *PKP2* frameshift mutation underlying ACM and having far-reaching implications on cardiac management of the family members including follow-up, lifestyle modification, medical treatment, and outcome. Such analysis requires highly specialized and highly trained multi-disciplinary care teams and should therefore be performed in dedicated cardiogenetics clinics.

## Data Availability Statement

The data presented in this study are available on request from the corresponding author. Data can also be found here https://databases.lovd.nl/shared/variants/0000708305#00001012.

## Ethics Statement

The studies involving human participants were reviewed and approved by IRCCS Fondazione Casa Sollievo della Sofferenza ethics committee. The patients/participants provided their written informed consent to participate in this study.

## Author Contributions

ML and PP contributed to conception of the manuscript, data collection and interpretation, drafting the article. JS contributed to critical revision and final approval of the version to be published. SM and GDS contributed to conception of the manuscript, data collection and interpretation, drafting the article, critical revision and final approval of the version to be published. SC and TM contributed to data collection, interpretation, and drafting the article. CA and DP contributed to drafting the article and critical revision. AP contributed to drafting the article. MCas contributed to conception of the manuscript. MCar contributed to conception of the manuscript, critical revision and final approval of the version to be published. All authors contributed to the article and approved the submitted version.

## Online Repository

https://databases.lovd.nl/shared/variants/0000708305#00001012

## Conflict of Interest

The authors declare that the research was conducted in the absence of any commercial or financial relationships that could be construed as a potential conflict of interest.

## Abbreviations

ACM: arrhythmogenic cardiomyopathy
ECG: electrocardiogram
FDRs: first degree relatives
NGS: next-generation sequencing
SCD: sudden cardiac death.
